# MoS_2_/NiSe_2_/rGO Multiple-Interfaced Sandwich-like Nanostructures as Efficient Electrocatalysts for Overall Water Splitting

**DOI:** 10.3390/nano13040752

**Published:** 2023-02-16

**Authors:** Xiaoyan Bai, Tianqi Cao, Tianyu Xia, Chenxiao Wu, Menglin Feng, Xinru Li, Ziqing Mei, Han Gao, Dongyu Huo, Xiaoyan Ren, Shunfang Li, Haizhong Guo, Rongming Wang

**Affiliations:** 1 Key Laboratory of Materials Physics, School of Physics and Microelectronics, Zhengzhou University, Ministry of Education, Zhengzhou 450052, China; 2 Beijing Advanced Innovation Center for Materials Genome Engineering, Beijing Key Laboratory for Magneto-Photoelectrical Composite and Interface Science, School of Mathematics and Physics, University of Science and Technology Beijing, Beijing 100083, China

**Keywords:** nanoparticle-doped bilayer-like nanostructures, multiple interfaces, overall water splitting, dual function, non-precious metal catalysis

## Abstract

Constructing a heterogeneous interface using different components is one of the effective measures to achieve the bifunctionality of nanocatalysts, while synergistic interactions between multiple interfaces can further optimize the performance of single-interface nanocatalysts. The non-precious metal nanocatalysts MoS_2_/NiSe_2_/reduced graphene oxide (rGO) bilayer sandwich-like nanostructure with multiple well-defined interfaces is prepared by a simple hydrothermal method. MoS_2_ and rGO are layered nanostructures with clear boundaries, and the NiSe_2_ nanoparticles with uniform size are sandwiched between both layered nanostructures. This multiple-interfaced sandwich-like nanostructure is prominent in catalytic water splitting with low overpotential for oxygen evolution reaction (OER) and hydrogen evolution reaction (HER) and almost no degradation in performance after a 20 h long-term reaction. In order to simulate the actual overall water splitting process, the prepared nanostructures are assembled into MoS_2_/NiSe_2_/rGO||MoS_2_/NiSe_2_/rGO modified two-electrode system, whose overpotential is only 1.52 mV, even exceeded that of noble metal nanocatalyst (Pt/C||RuO_2_~1.63 mV). This work provides a feasible idea for constructing multi-interface bifunctional electrocatalysts using nanoparticle-doped bilayer-like nanostructures.

## 1. Introduction

Since limited fossil energy is massively consumed and the resulting environmental pollution is becoming increasingly serious, the development of renewable and clean energy is a proven method and has become the future development trend [1,2,3,4]. The high-quality H_2_ produced in the hydrogen evolution reaction (HER) at the cathode of a water splitting device can be used as a green energy source, but the oxygen evolution reaction (OER) at the anode faces problems such as multiple reaction steps and slow kinetics [5,6,7]. Many efforts have been made to explore efficient electrocatalysts that can reduce the overpotential of both HER and OER to improve their reaction efficiency. However, the optimal catalysts for HER are Pt or Pt-based nanomaterials, differing from Ir- or Ru-based electrocatalysts for OER [8,9,10,11]. When completely different catalyst materials are used for the cathode and anode of a given water splitting device, the inconsistency of the two electrodes will lead to inefficiencies [12,13,14]. Accordingly, employing different components of nanocatalysts to construct heterogeneous interfaces is the most effective measure to alleviate the above problems, but it still faces a technical bottleneck to further improve the catalytic performance. In addition to searching for bifunctional catalysts that can catalyze both HER and OER in industrial applications, it is also crucial to find inexpensive materials to replace the high-cost Pt-, Ir-, and Ru-based electrocatalysts [15,16].

With the advantages of low cost, abundant reserves, and suitable d-electron configuration, transition metal-based catalysts have attracted much attention and are considered promising candidate materials for electrocatalysis [10,11,17]. Transition metal dichalcogenides (TMDs), represented by layered MoS_2_, are clearly the star players among them [18,19,20]. Early in 2005, Nørskov’s group explored the HER mechanism of different catalysts through density functional theory (DFT) calculations and found that the free energy of binding hydrogen atoms to MoS_2_ is similar to that of binding hydrogen atoms to Pt in HER, predicting that MoS_2_ is a promising non-noble metal catalyst [21]. However, the poor conductivity and inert planes of MoS_2_ lead to its suboptimal performance in electrocatalysis [22]. Graphene seems to compensate for the above-mentioned defects of MoS_2_ due to its ultra-thin nanostructure, large specific surface, and high electrical conductivity [23,24]. The combination of the two-layered nanostructures can expose abundant edge sites and provide favorable HER catalytic performance [25,26]. However, OER requires a higher overpotential than HER due to the involvement of a four-electron transfer process. Therefore, it is necessary to prepare an excellent electrocatalyst to accelerate the kinetics and reduce the overpotential in the OER process for obtaining the dual function of water splitting.

The selenides featuring the 4s^2^p^4^ electronic structure and vacant 3d orbitals can form strong metal bonds with transition metals, and this is very profitable for the multi-electron transport of OER [27]. Due to the narrow band gap (≈2.0 eV) of NiSe_2_ and the good corrosion resistance of Ni in alkaline solvents, NiSe_2_ has been identified as a very important non-precious metal OER catalyst in alkaline media [28]. Park et al. compared the catalytic performance of NiSe_2_ and CoSe_2_ for OER and found that the former was superior to the latter due to the oxide layer in NiSe_2_ as the actual active site was more active than that in CoSe_2_ [29]. Xu et al. proposed a strategy of heteroatom-doped composite phase engineering to further modulate the electronic conductivity and active sites of NiSe_2_ to obtain more excellent OER properties [30]. With this understanding, the introduction of nanoparticles (NiSe_2_) in bilayer nanomaterials (MoS_2_/reduced graphene oxide (rGO)) to build the multi-heterogeneous interfaces and create the bifunctional catalysts seems to offer an exciting approach for catalytic water splitting.

Based on the above issues, a MoS_2_/NiSe_2_/rGO multiple interfaced sandwich-like nanostructure was synthesized by a simple hydrothermal method, where MoS_2_ and rGO are layered nanostructures with clear boundaries and NiSe_2_ are nanoparticles with a diameter of about 20 nm. The obtained MoS_2_/NiSe_2_/rGO nanostructures exhibited extremely high electrochemical activity for both OER and HER in alkaline electrolytes by taking advantage of the synergistic effect of the three interfaces (MoS_2_/NiSe_2_, NiSe_2_/rGO, and MoS_2_/rGO). This approach of doping TMD nanoparticles in the middle of bilayer-like nanostructures to obtain multiple interfaces can provide a new strategy for obtaining efficient bifunctional catalysts.

## 2. Materials and Methods

### 2.1. Materials

NiCl_2_·6H_2_O, (NH_4_)_6_Mo_7_O_24_·4H_2_O, and polytetrafluoroethylene (PTFE, 10%) are purchased from Aladdin Ltd. (Shanghai, China). NaBH_4_ and L-cysteine are purchased from Kermel (Tianjin, China). Se powder (99.9%) is purchased from Xiya Reagent (Linyi, China). KOH and Ruthenium Dioxide (RuO_2_) are purchased from Macklin (Shanghai, China). JM 40% Pt/C catalyst is purchased from Hesen (Shanghai, China). Ni foam is purchased from Changde Liyuan New Materials Co. Ltd. (Changde, China). Ethanol and acetone are purchased from Hengxing (Tianjin, China). All reagents are purchased ready for use without further processing.

### 2.2. Synthesis of NiSe_2_/rGO

Graphene oxide (GO) is prepared from pristine graphite flakes using an improved Hummers’ method [31]. NiSe_2_/rGO is prepared by an improved facile hydrothermal process [32]. First, 10 mmol NaBH_4_ and 8 mmol Se powder are dispersed in 50 mL deionized water with vigorous magnetic stirring for 1 h at room temperature. At the same time, 25 mg GO is dispersed in 20 mL deionized water by ultrasonic treatment for 30 min, and then 4 mmol NiCl_2_·6H_2_O is added, and the ultrasonic treatment is continued for 30 min. Subsequently, the Se-containing solution and GO-containing suspension are mixed and stirred for 1 h. After that, the mixture is transferred into a 100 mL Teflon-lined autoclave for hydrothermal treatment at 160 °C for 12 h. The resulting product (NiSe_2_/rGO) is collected by filtration, washed with deionized water and ethanol several times, and then dried at 60 °C. For comparison, bare NiSe_2_ catalyst is prepared in a similar procedure without adding GO.

### 2.3. Preparation of MoS_2_/NiSe_2_/rGO

The MoS_2_/NiSe_2_/rGO composite is prepared by the second hydrothermal process by improving the reported MoS_2_ preparation method [33]. Total amounts of 130 mg NiSe_2_/rGO, 100 mg (NH_4_)_6_Mo_7_O_24_·4H_2_O, and 290 mg L-cysteine are dispersed in 30 mL deionized water with vigorous magnetic stirring at room temperature for 30 min. Then, the mixture is transferred into a 50 mL Teflon-lined autoclave for hydrothermal treatment at 220 °C for 24 h. The resulting product (MoS_2_/NiSe_2_/rGO) is collected by filtration, washed with deionized water and ethanol several times, and then dried at 60 °C. For comparison, the mass of (NH_4_)_6_Mo_7_O_24_·4H_2_O is changed to 50 and 150 mg, and other preparation conditions are kept constant to obtain MoS_2_/NiSe_2_/rGO-1 and MoS_2_/NiSe_2_/rGO-2, respectively. Similarly, other comparison samples are obtained: MoS_2_, MoS_2_/NiSe_2_, MoS_2_/NiSe_2_-1, and MoS_2_/NiSe_2_-2.

### 2.4. Morphological and Structural Characterization

The morphology of MoS_2_/NiSe_2_/rGO is obtained using a focused ion beam scanning electron microscopy (SEM; JCM-6000PLUS, JEOL, Tokyo, Japan) and transmission electron microscopy (TEM; JEM-2100, JEOL, Tokyo, Japan). The structure of MoS_2_/NiSe_2_/rGO is analyzed using a powder X-ray diffractometer (XRD; Smart Lab SE, Rigaku, Osaka, Japan). The phase composition and the electronic structures of the elements are investigated via Raman spectroscopy (Raman; Renishaw inVia, Renishaw, New Mills, UK) and X-ray photoelectron spectroscopy (XPS; AXIS Supra, Kratos, Manchester, UK).

### 2.5. Electrocatalysis Measurements

An amount of 20.0 mg of MoS_2_/NiSe_2_/rGO material is dispersed in a 1.0 mL mixed solution of ethanol/deionized water/10% PTFE (1:1:1), ultrasonically treated for 10 min to form a uniform suspension, and then dropped onto clean Ni foam (NF), followed by drying under vacuum at 60 °C. The final mass load of MoS_2_/NiSe_2_/rGO material is calculated to be 4.0 mg cm^−2^.

The electrochemical measurements of OER and HER are measured using a three-electrode workstation (CHI660E, Shanghai Chenhua Instruments, Shanghai, China) at room temperature. The counter, reference, and working electrodes are graphite rods, Ag/AgCl electrodes, and Ni foam electrodes, respectively. The tested potentials are calibrated to reversible hydrogen electrode (RHE) by the Nernst equation: E_vs.RHE_ = E_vs.Ag/AgCl_ + 0.059 pH + 0.197, all linear sweep voltammetry (LSV) curves are corrected with IR compensation (LSV-IR) derived from the electrochemical impedance spectroscopy (EIS) measurements under open circuit voltage [34]. The electrochemical double layer capacitance (C_dl_) of the catalyst can be obtained from the linear slope between the current density difference at the cathode and anode and the scan rate [27]. The overall water splitting measurements are measured using a two-electrode system in 1.0 M KOH solution.

## 3. Results

**Figure 1:**
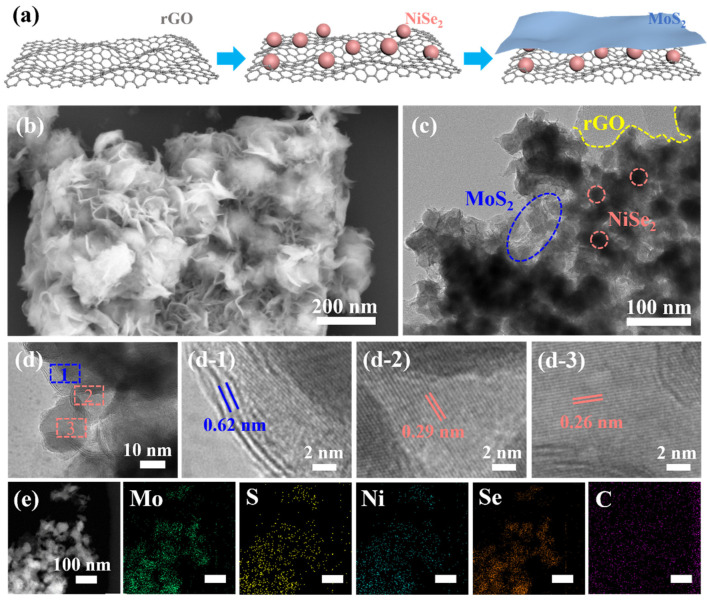
Structural characterizations of MoS_2_/NiSe_2_/rGO. (**a**) Schematic illustration of the MoS_2_/NiSe_2_/rGO formation process. (**b**) SEM, (**c**) TEM, (**d**) HRTEM, and (**e**) HADDF-STEM images and the corresponding element mapping of MoS_2_/NiSe_2_/rGO. (**d-1**,**d-2**,**d-3**) are the local enlargements of (**d**), respectively.

The improved Hummers’ method is used to prepare rGO with good quality, exhibiting a flat surface and the expected ductility, as shown in Figure S1. Then, the NiSe_2_ nanoparticles and MoS_2_ layered nanostructures with different ratios are sequentially grown on rGO by a simple hydrothermal method to obtain a series of MoS_2_/NiSe_2_/rGO composites. A schematic diagram of the MoS_2_/NiSe_2_/rGO fabrication process is shown in Figure 1a. Figure S2 shows the SEM images of NiSe_2_ and NiSe_2_/rGO, respectively, and it can be seen that the uniformly sized NiSe_2_ nanoparticles do not change their size before and after being anchored on rGO. According to the designed plan, MoS_2_ is deposited on NiSe_2_/rGO in order to obtain the MoS_2_/NiSe_2_/rGO bilayer-like sandwich nanostructures. It can be seen from Figure 1b and Figure S3 that after loading MoS_2_, both MoS_2_ and NiSe_2_ maintain their previous morphology. Notably, the MoS_2_ layered nanostructure becomes more and more dense as the amount of MoS_2_ increases by keeping NiSe_2_ invariant, as shown in Figures S4b–d. However, after they are loaded on rGO (Figure 1b and Figure S4e,f), this denseness decreases, and the dispersibility increases, indicating that rGO can be used as a good dispersion substrate.

**Figure 2:**
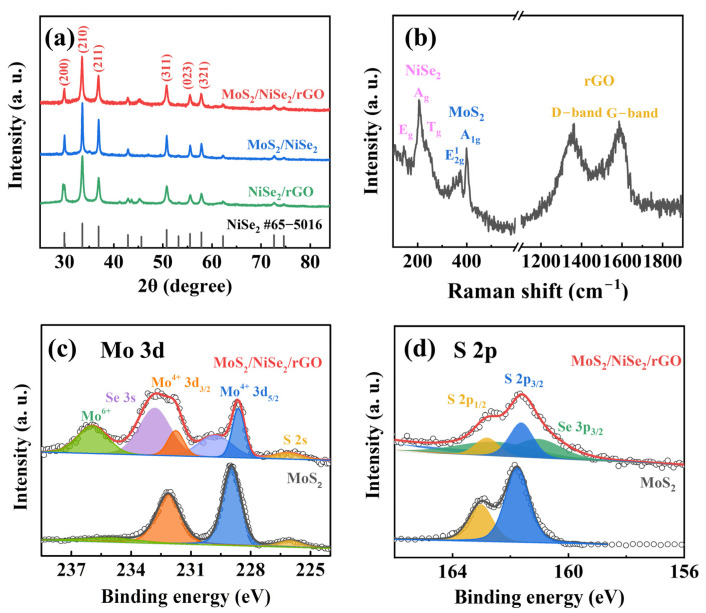
Chemical analysis of the MoS_2_/NiSe_2_/rGO. (**a**) XRD patterns of MoS_2_/NiSe_2_/rGO, MoS_2_/NiSe_2_, and NiSe_2_/rGO. (**b**) Raman spectra of MoS_2_/NiSe_2_/rGO. XPS spectra of (**c**) Mo 3d and (**d**) S 2p for MoS_2_ and MoS_2_/NiSe_2_/rGO.

**Figure 3:**
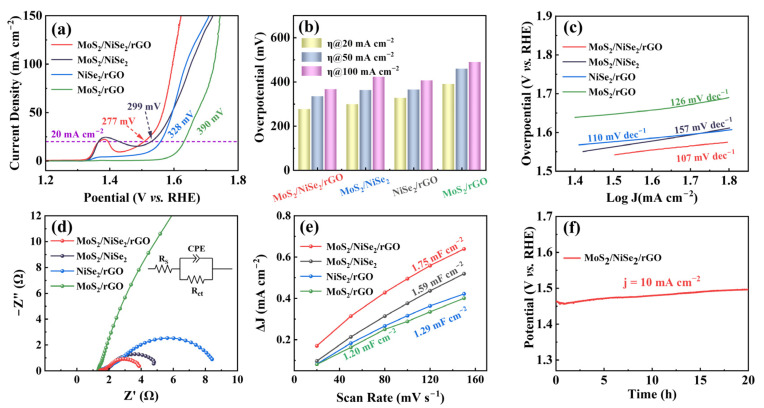
The OER performance of MoS_2_/NiSe_2_/rGO, MoS_2_/NiSe_2_, NiSe_2_/rGO, and MoS_2_/rGO in 1.0 M KOH solution. (**a**) Polarization curves (iR-corrected) at a scan rate of 2 mV s^−1^. (**b**) Overpotential required at a current density of 20, 50, and 100 mA cm^−2^. (**c**) Tafel plots. (**d**) Nyquist plots at an overpotential of 300 mV and fitted lines for an equivalent circuit. (**e**) The C_dl_ values can be estimated from the linear slopes between ∆j (=j_anodic_ − j_cathodic_) and scan rates. (**f**) Chronopotentiometry curve of MoS_2_/NiSe_2_/rGO with a constant current density of 10 mA cm^−2^.

The structure of MoS_2_/NiSe_2_/rGO is characterized more finely by TEM, and it is clearly seen in Figure 1c that MoS_2_ and rGO have a high-quality layered structure with NiSe_2_ nanoparticles uniformly sandwiched between both, forming a well-defined bilayer-like sandwich nanostructure. Figure 1d shows a typical local structure at the boundary, and Figure 1(d-1,d-2,d-3) are enlarged views of the boxes therein, respectively. Clear lattice stripes with spacings of 0.62, 0.29, and 0.26 nm are visible in these three enlarged images, corresponding to the (002) plane of MoS_2_ and the (200) and (210) planes of NiSe_2_, respectively [18,35]. The STEM-energy dispersive spectrometer (EDS) elemental mapping of MoS_2_/NiSe_2_/rGO in Figure 1e confirms the presence of element C and shows its uniform distribution in the layered sandwich-like nanostructure. It is also noted that the distributions of Mo, S, Ni, and Se elements are almost identical, indicating not only that MoS_2_ and NiSe_2_ are uniformly distributed on rGO but also that the NiSe_2_ nanoparticles are covered in the MoS_2_ layered nanostructure.

Subsequently, the structural characteristics, phase composition, and element analysis of the prepared samples are analyzed by XRD, Raman, and XPS spectra, respectively. Figures S1b and S5a,b exhibit the XRD spectra of rGO, NiSe_2_, and MoS_2_, respectively. As shown in Figure 2a, distinct diffraction peaks appear at 29.67°, 33.61°, 36.95°, 50.79°, 55.58°, and 57.82° in MoS_2_/NiSe_2_/rGO, MoS_2_/NiSe_2_, and NiSe_2_/rGO, corresponding to the (200), (210), (211), (311), (023), and (321) crystal plane of NiSe_2_, respectively, which are well consistent with the standard crystal of NiSe_2_ (PDF No. 65-5016), indicating that NiSe_2_ exhibits excellent crystallinity in all three samples [35]. In contrast, none of the diffraction peaks of MoS_2_ appear, which is due to the poor crystallinity of MoS_2_ obtained by the hydrothermal method [36]. Raman spectroscopy is used to further determine the composition of MoS_2_/NiSe_2_/rGO. As shown in Figure 2b and Figure S6, the Raman characteristic peaks of the MoS_2_/NiSe_2_/rGO and pure MoS_2_ samples at 375 and 401 cm^−1^ belong to the $E_{2g}^{1}$ and A_1g_ modes of the semiconductor phase (2H) MoS_2_, representing the in-plane Mo–S bond and the out-of-plane S atom vibration mode, respectively [37,38]. In addition, the characteristic peaks of NiSe_2_ are also displayed at 148, 208, and 235 cm^−1^, while the peaks at 1358 and 1588 cm^−1^ correspond to the D band and G band of rGO, respectively [39,40]. The Raman results confirm that the 2H-phase-dominated MoS_2_ nanosheets are on the NiSe_2_/rGO surfaces.

XPS is used to examine the molecular structure and atomic valence states of the materials [41]. The presence of Mo, S, Ni, and Se in the MoS_2_/NiSe_2_/rGO material is confirmed from the full XPS spectrum (Figure S7a), but the Ni content on the surface of the sample is relatively low (Figure S7b). Figure 2c,d present the high-resolution XPS spectra of Mo 3d and S 2p in pure MoS_2_ and MoS_2_/NiSe_2_/rGO, respectively. As shown in Figure 2c, the fitting peaks of the pure MoS_2_ at 232.2, 229, and 226.1 eV correspond to Mo^4+^ 3d_3/2_, Mo^4+^ 3d_5/2_, and S 2s, respectively, while the high binding energy peak of the Mo 3d (235.5 eV) corresponds to the MoO_3_, which may result from the oxidation of the sample in the air [42]. Compared with pure MoS_2_, both the peaks of Mo^4+^ 3d_3/2_ and Mo^4+^ 3d_5/2_ in MoS_2_/NiSe_2_/rGO are slightly shifted to lower binding energies (~0.35 eV), which indicates that there is a significant electron transfer between NiSe_2_ and MoS_2_. In addition, the peak at 232.8 eV is attributed to Mo-Se, which also indicates the formation of a heterogeneous interface between MoS_2_ and NiSe_2_, where some of the Se atoms replace the S atoms [43].

As shown in the XPS spectrum of S 2p (Figure 2d), pure MoS_2_ has only two typical peaks corresponding to S 2p_1/2_ and S 2p_3/2_. In contrast, the S_Mo-S_ 2p_1/2_ and S_Mo-S_ 2p_3/2_ of the MoS_2_/NiSe_2_/rGO have a negative offset, implying the occurrence of electron transfer. At the same time, two peaks are separated at 161.6 and 162.8 eV, corresponding to S_Ni-S_ 2p_1/2_ and S_Ni-S_ 2p_3/2_, respectively. Compared with pure MoS_2_, the formation of heterostructure caused a slight negative shift in the energy band position of S (~0.16 eV), which is due to the synergistic effect between NiSe_2_ and molybdenum disulfide [44]. In addition, the positive displacement of Se 3d in Figure S7c fully proves this point. The XPS results confirmed the strong interfacial electronic interactions in the heterostructure and the electrons transfer from NiSe_2_ to MoS_2_, which would optimize the electronic structure of NiSe_2_ and MoS_2_ and thus enhance the activity of OER and HER.

The OER electrocatalytic performance of MoS_2_/NiSe_2_/rGO in an alkaline solution (1 M KOH) is first investigated (Figure 3). To better demonstrate the effect of multiple interfaces on performance, three heterogeneous interfaces (MoS_2_/NiSe_2_, NiSe_2_/rGO, and MoS_2_/rGO) consisting of three constituent units are used as the samples to be tested under the same conditions. Meanwhile, the properties are tested for NiSe_2_, NF, and RuO_2_, and different precursor ratios of MoS_2_/NiSe_2_-1, MoS_2_/NiSe_2_-2, MoS_2_/NiSe_2_/rGO-1, and MoS_2_/NiSe_2_/rGO-2 are investigated, as shown in Figure S8. From the iR-compensated LSV curves (Figure 3a), it is obvious that MoS_2_/NiSe_2_/rGO has the optimum performance among the four electrocatalysts. When the current density is 20 mA cm^−2^, the overpotential of MoS_2_/NiSe_2_/rGO is only 277 mV, which is 22, 51, and 113 mV lower than that of MoS_2_/NiSe_2_, NiSe_2_/rGO, and MoS_2_/rGO, respectively. For a comprehensive comparison of the LSV performance of the four catalysts, whose overpotentials at two additional current densities are also identified. As shown in Figure 3b, although the overpotentials of the four catalysts increase gradually with the current density, the overpotential of MoS_2_/NiSe_2_/rGO is consistently the lowest among them. The overpotential of MoS_2_/NiSe_2_/rGO is surprisingly 123 mV lower than that of MoS_2_/rGO when the current density is 100 mA cm^−2^, and the relevant values are shown in Table S2. The results fully demonstrate that the NiSe_2_ nanoparticles sandwiched between the two layered structures can effectively optimize the overpotential of MoS_2_/rGO. Furthermore, multiple interfacial nanostructures can greatly improve the contribution of a single interface to performance, and this new multi-interfacial nanostructure constructed by the three composites acting together can facilitate the OER performance [44,45].

The enhanced OER kinetics of MoS_2_/NiSe_2_/rGO is confirmed by the smaller Tafel slope (107 mV dec^−1^) compared to those of MoS_2_/NiSe_2_ (157 mV dec^−1^), NiSe_2_/rGO (110 mV dec^−1^), and MoS_2_/rGO (126 mV dec^−1^), as shown in Figure 3c. To further validate the electron transfer dynamics of OER, EIS are also performed on four electrocatalysts. From the corresponding Nyquist plots in Figure 3d, it can be seen that MoS_2_/NiSe_2_/rGO exhibits significantly lower charge transfer resistance compared to NiSe_2_/rGO, MoS_2_/NiSe_2_, and MoS_2_/rGO, indicating faster electron transfer on MoS_2_/NiSe_2_/rGO, and also suggesting that the multiple interfaced sandwich-like nanostructure can significantly improve the conductivity of the electrocatalyst. The C_dl_ values are proportional to the electrochemical active surface area (ECSA) and can be estimated by performing cyclic voltammetry (CV) curves at different scan rates in a non-faradic potential range, which is usually used to further evaluate the OER catalytic activity [11]. As shown in Figure 3e, the C_dl_ values of the four electrocatalysts are calculated from the slopes of the linear fits of the CV curves (Figure S9), in which MoS_2_/NiSe_2_/rGO has the largest electrical double-layer capacitance value, denoting a larger electrochemically active surface area. Durability is also an important indicator to assess the performance of the electrocatalyst. The chronopotential measurement curve of MoS_2_/NiSe_2_/rGO at a constant current density of 10 mA cm^−2^ is obtained for continuous 20 h (Figure 3f). Compared to the initial potential of 1.46 V, there is only a slight elevation of 30 mV in the potential after 20 h of continuous oxygen generation. This indicates that the as-prepared MoS_2_/NiSe_2_/rGO not only has excellent catalytic activity, but also has outstanding catalytic durability.

**Figure 4:**
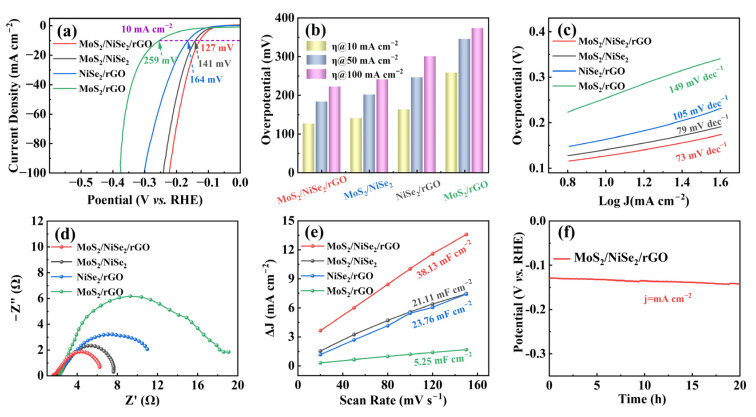
HER performance of the MoS_2_/NiSe_2_/rGO, MoS_2_/NiSe_2_, NiSe_2_/rGO, and MoS_2_/rGO in 1.0 M KOH solution. (**a**) Polarization curves (iR-corrected) at a scan rate of 5 mV s^−1^. (**b**) Overpotential required at a current density of 10, 50, and 100 mA cm^−2^. (**c**) Tafel plots. (**d**) Nyquist plots at an overpotential of 120 mV. (**e**) The C_dl_ values can be estimated from the linear slopes between ∆j (=j_anodic_ − j_cathodic_) and scan rates. (**f**) Chronopotentiometry curve of MoS_2_/NiSe_2_/rGO with a constant current density of 10 mA cm^−2^.

The HER performance of the samples is evaluated in the same electrolyte (1.0 M KOH) at a scan rate of 5 mV s^−1^ (Figure 4 and Figure S10). Figure 4a shows the LSV curves of MoS_2_/NiSe_2_/rGO, MoS_2_/NiSe_2_, NiSe_2_/rGO, and MoS_2_/rGO with iR-compensation, and it can be seen that the curves are smooth with a uniform trend, which proves that all four catalysts have HER activity. However, the overpotential of the four catalysts differed greatly at the same current density, as marked in the purple line in Figure 4a at a current density of 10 mA cm^−2^, and MoS_2_/NiSe_2_/rGO has the lowest overpotential among the four catalysts, indicating that it has the favorable HER activity. For a more comprehensive assessment of the intrinsic reaction kinetics of the catalysts, the overpotentials at different current densities are visualized in Figure 4b as histograms. It can be seen that MoS_2_/NiSe_2_/rGO exhibits the lowest overpotential regardless of the determined current density and is the best among four samples in HER. Even at a high current density of 100 mA cm^−2^, its overpotential is only 223 mV, which is better than 241 mV of MoS_2_/NiSe_2_, 301 mV of NiSe_2_/rGO, and 374 mV of MoS_2_/rGO.

The calculated Tafel slopes of the four catalysts are calculated to be 73, 79, 105, and 149 mV dec^−1^, respectively, as shown in Figure 4c. The lower Tafel slopes of MoS_2_/NiSe_2_/rGO and MoS_2_/NiSe_2_ prove that the combination of MoS_2_ and NiSe_2_ helps to improve the reaction rate and kinetics [46]. The corresponding Nyquist plots shown in Figure 4d are similar to the OER test results. Compared with MoS_2_/NiSe_2_, NiSe_2_/rGO, and MoS_2_/rGO, MoS_2_/NiSe_2_/rGO exhibits lower charge transfer resistance, indicating that MoS_2_/NiSe_2_/rGO has superior electron transport capability. In addition, the C_dl_ is again used to assess the HER catalytic activity of the catalysts. Figure 4e and Figure S11 reveal that the fitted line slopes of 38.13, 21.11, 23.76, and 5.25 mF cm^−2^ for MoS_2_/NiSe_2_/rGO, MoS_2_/NiSe_2_, NiSe_2_/rGO, and MoS_2_/rGO, respectively, suggesting that the multiple interfaced sandwich-like nanostructure provides more effective active sites than the other three catalysts [47]. Of these, more active sites might be due to the effect of the unique heterogeneous interface of MoS_2_/NiSe_2_/rGO. Furthermore, the HER electrochemical stability of MoS_2_/NiSe_2_/rGO is also evaluated by the chronopotentiometry measurement curve (Figure 4f). After 20 h of continuous testing, the overpotential increases by only 15 mV at a constant current density of 10 mA cm^−2^. Moreover, MoS_2_/NiSe_2_/rGO shows negligible catalytic degradation, confirming its excellent electrochemical stability.

**Figure 5:**
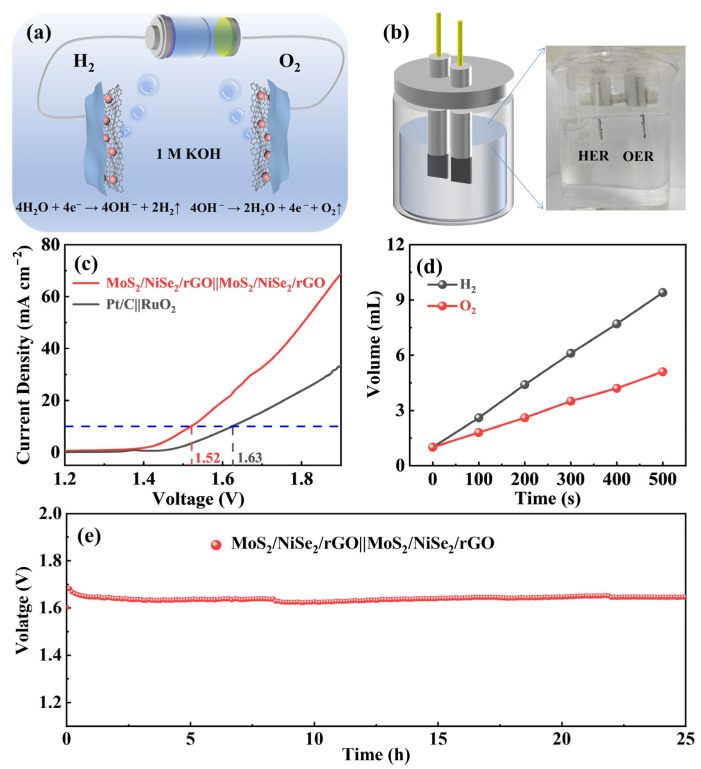
Overall water splitting performance of MoS_2_/NiSe_2_/rGO in 1.0 M KOH solution. (**a**) Schematic diagram of overall water splitting. (**b**) Double-electrode device for overall water splitting. (**c**) LSV curves of the MoS_2_/NiSe_2_/rGO||MoS_2_/NiSe_2_/rGO and Pt/C||RuO_2_ catalysts. Inset: A photo of the overall water splitting cell. (**d**) The production of H_2_ and O_2_ as a function of time. (**e**) Time-dependent current density curve of MoS_2_/NiSe_2_/rGO||MoS_2_/NiSe_2_/rGO for 25 h.

In view of the excellent OER and HER catalytic behaviors of the MoS_2_/NiSe_2_/rGO catalyst discussed above, it is further served as a bifunctional catalyst and assembled into a two-electrode test system to investigate its overall water splitting performance in 1.0 M KOH electrolyte. The schematic diagram of all-water electrolysis in the double-electrode test system is shown in Figure 5a. At the cathode, the electrons transferred to the electrolyte combine with water to produce H_2_ gas, while the generated hydroxide ions are transferred to the anode and oxidized to release O_2_ gas. Polarized electrons are released at the anode so that the hydroxide is oxidized in the solution to release O_2_ gas. The two-electrode test device is shown in Figure 5b. In the LSV curves with a scan rate of 5 mV s^−1^ shown in Figure 5c, MoS_2_/NiSe_2_/rGO||MoS_2_/NiSe_2_/rGO exhibits excellent catalytic activity, affording a current density of 10 mA cm^−2^ at 1.52 V, while Pt/C||RuO_2_ requires the same current density at 1.63 V. The inset is a photograph of the experimental device, clearly showing the high density of bubbles on the surface of both electrodes. The H_2_ and O_2_ generated from the MoS_2_/NiSe_2_/rGO catalyzed overall water-splitting are collected quantitatively by the drainage method. As shown in Figure 5d, the volume ratio of collected H_2_ to O_2_ is 2.05:1, close to the theoretical value of 2:1. Taking into account that the airtightness of the equipment produces errors, it can be seen that the Faraday efficiency of the overall water splitting is almost 100% [48]. The stability test results of MoS_2_/NiSe_2_/rGO at a constant current density of 10 mA cm^−2^ are shown in Figure 5e. It can be seen from the chronopotential curve that MoS_2_/NiSe_2_/rGO||MoS_2_/NiSe_2_/rGO features extremely outstanding stability, which maintains the output voltage almost constant at 1.65 V throughout the continuous 25 h of the overall water splitting performance. In general, MoS_2_/NiSe_2_/rGO is proven to trigger the overall water splitting with favorable efficiency and stability. Compared with the recently reported advanced electrocatalysts, MoS_2_/NiSe_2_/rGO demonstrates outstanding performance (Table S1), indicating that the prepared sandwich nanostructure is an excellent overall water splitting bifunctional electrocatalyst [16,49,50,51,52,53].

The outstanding OER, HER, and overall water splitting performance of MoS_2_/NiSe_2_/rGO, where OER and overall water splitting even surpasses those of noble metals (Figure 5 and Figure S8), are mainly attributed to the highly advantageous multiple interfaced sandwich-like nanostructure of the NiSe_2_ nanoparticles sandwiched between two different layered structures (MoS_2_ and rGO). The DFT calculations confirm this point of view (Figure S12). Due to the smaller size of the intercalated NiSe_2_ nanoparticles, this unique nanostructure can simultaneously construct three different interfacial relationships: MoS_2_/NiSe_2_, NiSe_2_/rGO, and MoS_2_/rGO, and the synergistic effect of the three interfaces can dramatically facilitate the rapid and smooth electrocatalytic reaction. Moreover, the NiSe_2_ nanoparticles are sandwiched between both layers, making them less tightly packed and contributing to the rapid transport of the gases generated by the reaction, enhancing the reaction dynamics, as evidenced by the Tafel curves. In addition, compared with the ordinary nanoparticles, both layered nanostructures that supported nanoparticles are more helpful in maintaining a robust nanostructure for excellent stability. This design approach can offer new ideas for obtaining efficient catalysts with structural stability.

## 4. Conclusions

In summary, MoS_2_/NiSe_2_/rGO with multiple interfaced sandwich-like nanostructures consisting of NiSe_2_ nanoparticles sandwiched between layered MoS_2_ and rGO are successfully prepared by a simple and convenient two-step hydrothermal method. Compared with similar catalysts, MoS_2_/NiSe_2_/rGO has better electrocatalytic performance, even OER and overall water splitting surpass those of the noble metals, which are attributed to the efficient interfacial relationship between MoS_2_/NiSe_2_, NiSe_2_/rGO, and MoS_2_/rGO. The overpotential of MoS_2_/NiSe_2_/rGO||MoS_2_/NiSe_2_/rGO in overall water splitting is only 1.52 mV, which is much lower than that of the noble metal electrocatalyst Pt/C||RuO_2_ (1.63 mV). Additionally, there is almost no loss of performance in the long-term stability test, implying its good stability. This multi-interface idea can open new avenues for the design of efficient bifunctional overall water catalysts.

## Data Availability

The data presented in this study are available on request from the corresponding author.

## Supplementary Materials

The following supporting information can be downloaded at: https://www.mdpi.com/article/10.3390/nano13040752/s1, Figure S1. (a) SEM image and (b) XRD pattern of the graphene oxide (GO); Figure S2. SEM images of (a) NiSe_2_ and (b) NiSe_2_/rGO; Figure S3. SEM image of the pure MoS_2_; Figure S4. SEM images of (a) NiSe_2_, (b) MoS_2_/NiSe_2_-1, (c) MoS_2_/NiSe_2_, (d) MoS2/NiSe_2_-2, (e) MoS_2_/NiSe_2_/rGO-1, and (f) MoS_2_/NiSe_2_/rGO-2; Figure S5. XRD patterns of (a) NiSe_2_, MoS_2_/NiSe_2_, MoS_2_/NiSe_2_-1, MoS_2_/NiSe_2_-2, MoS_2_/NiSe_2_/rGO, MoS_2_/NiSe_2_/rGO-1, MoS_2_/NiSe_2_/rGO-2 and (b) the pure MoS_2_; Figure S6. Raman spectra of NiSe_2_, NiSe_2_/rGO, MoS_2_/NiSe_2_, MoS_2_/NiSe_2_/rGO and MoS_2_; Figure S7. (a) XPS survey spectra. (b) Ni 2p and (c) Se 3d high resolution spectrum of MoS_2_/NiSe_2_/rGO, respectively; Figure S8. (a) Polarization curves (iR-corrected) at a scan rate of 2 mV s^−1^ of NiSe_2_, MoS_2_/NiSe_2_, MoS_2_/NiSe_2_-1, MoS_2_/NiSe_2_-2, MoS_2_/NiSe_2_/rGO, MoS_2_/NiSe_2_/rGO-1, MoS_2_/NiSe_2_/rGO-2, RuO_2_ and Ni foam, respectively. (The overall thickness, porosity, thread thickness, and pore density of Ni foam is 0.3 mm, 96%, 40–100 μm, and 110 PPI, respectively.) (b) Tafel plots of a series of samples. (c) Nyquist plots at an overpotential of 300 mV; Figure S9. CV curves for (a) MoS_2_/NiSe_2_/rGO, (b) MoS_2_/NiSe_2_, (c) NiSe_2_/rGO, (d) MoS_2_/rGO with various scan rates (20, 50, 80, 100, 120, 150 mV s^−1^); Figure S10. (a) Polarization curves (iR-corrected) at a scan rate of 5 mV s^−1^ of NiSe_2_, MoS_2_/NiSe_2_, MoS_2_/NiSe_2_-1, MoS_2_/NiSe_2_-2, MoS_2_/NiSe_2_/rGO, MoS_2_/NiSe_2_/rGO-1, MoS_2_/NiSe_2_/rGO-2, Pt/C and Ni foam, respectively. (b) Tafel plots of a series of samples. (c) Nyquist plots at an overpotential of 120 mV; Figure S11. CV curves for (a) MoS_2_/NiSe_2_/rGO, (b) MoS_2_/NiSe_2_, (c) NiSe_2_/rGO, (d) MoS_2_/rGO with various scan rates (20, 50, 80, 100, 120, 150 mV s^−1^); Figure S12. Optimized structural representations for hydrogen adsorption at (a) MoS_2_, (b) MoS_2_/rGO, (c) MoS_2_/NiSe_2_/rGO. Calculated free energy diagrams of the HER on MoS_2_, MoS_2_/rGO, NiSe_2_/rGO, and MoS_2_/NiSe_2_/rGO, respectively; Table S1. Comparison of electrocatalytic performance of different catalysts in 1 M KOH electrolyte; Table S2. Comparison of the electrocatalytic activity of MoS_2_/NiSe_2_/rGO with MoS_2_/rGO, NiSe_2_/rGO in 1 M KOH electrolyte. References [16,49,50,51,52,53,54,55,56,57] are cited in the Supplementary Materials.
