# Supplementary material for: MoS2/NiSe2/rGO Multiple-Interfaced Sandwich-like Nanostructures as Efficient Electrocatalysts for Overall Water Splitting
Source: Nanomaterials (Basel). 2023 Feb 16;13(4):752. doi: 10.3390/nano13040752 (PMC9964359; doi:10.3390/nano13040752)
Supplement: Supplementary file 1 [file nanomaterials-13-00752-s001.zip › nanomaterials-2200386-supplementary.pdf]

nanomaterials

MDPI

Supplementary Materials

$MoS_2/NiSe_2/rGO$ Multiple-Interfaced Sandwich-like Nanostructures as Efficient Electrocatalysts for Overall Water Splitting

Xiaoyan Bai [1,†], Tianqi Cao [1,†], Tianyu Xia [1,*], Chenxiao Wu [1], Menglin Feng [1], Xinru Li [1], Ziqing Mei [1], Han Gao [1], Dongyu Huo [1], Xiaoyan Ren [1], Shunfang Li [1], Haizhong Guo [1,*] and Rongming Wang [2,*]

1 Key Laboratory of Materials Physics, Ministry of Education, School of Physics and Microelectronics, Zhengzhou University, Zhengzhou 450052, China; xy11abai@163.com (X.B.); tqcao123@163.com (T.C.); w_chenxiao@163.com (C.W.); fengmenglin0126@163.com (M.F.); xinrulizk@163.com (X.L.); mzq6667@163.com (Z.M.); hgao@zzu.edu.cn (Han Gao); huodongyu@gs.zzu.edu.cn (D.H.); renxyan@zzu.edu.cn (X.R.); sflizzu@zzu.edu.cn (S.L.)

2 Beijing Advanced Innovation Center for Materials Genome Engineering, Beijing Key Laboratory for Magneto-Photoelectrical Composite and Interface Science, School of Mathematics and Physics, University of Science and Technology Beijing, Beijing 100083, China

* Correspondence: tyxia@zzu.edu.cn (T.X.); hguo@zzu.edu.cn (Haizhong Guo); rmwang@ustb.edu.cn (R.W.)

† These authors contributed equally to this work.

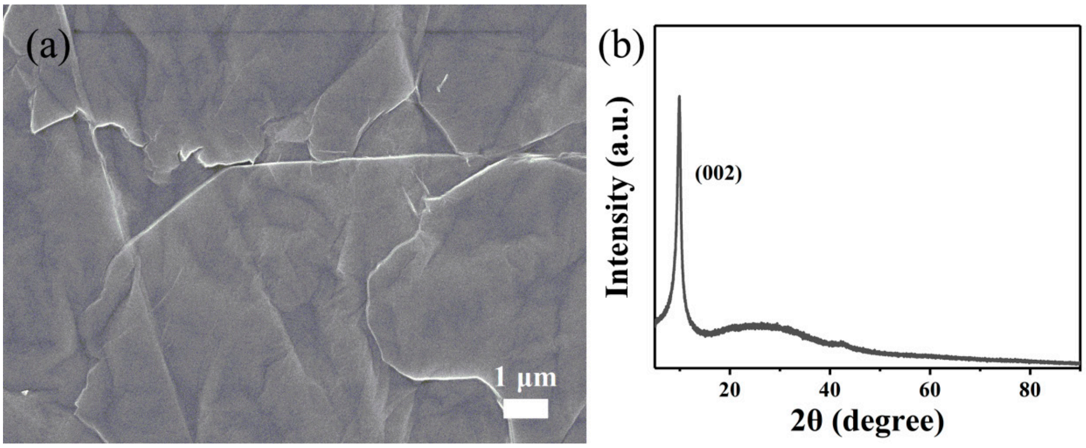

Figure S1. (**a**) SEM image and (**b**) XRD pattern of the graphene oxide (GO).

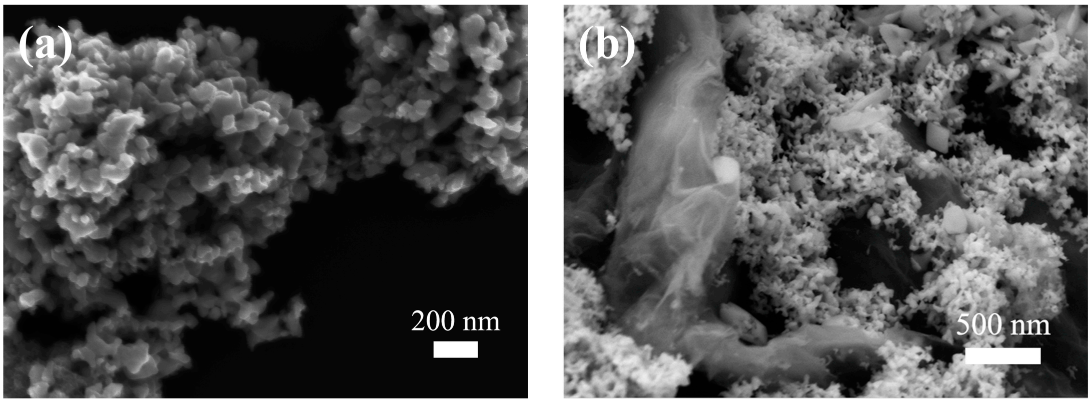

Figure S2. SEM images of (**a**) $NiSe_2$ and (**b**) $NiSe_2$/rGO.

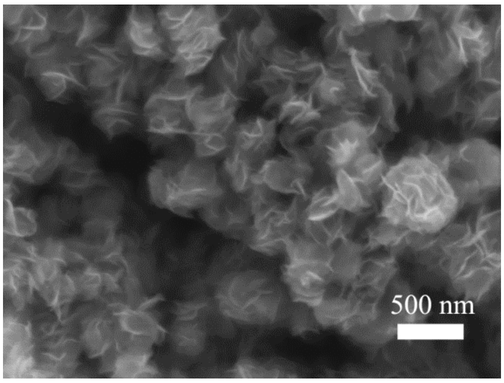

Figure S3. SEM image of the pure MoS_2.

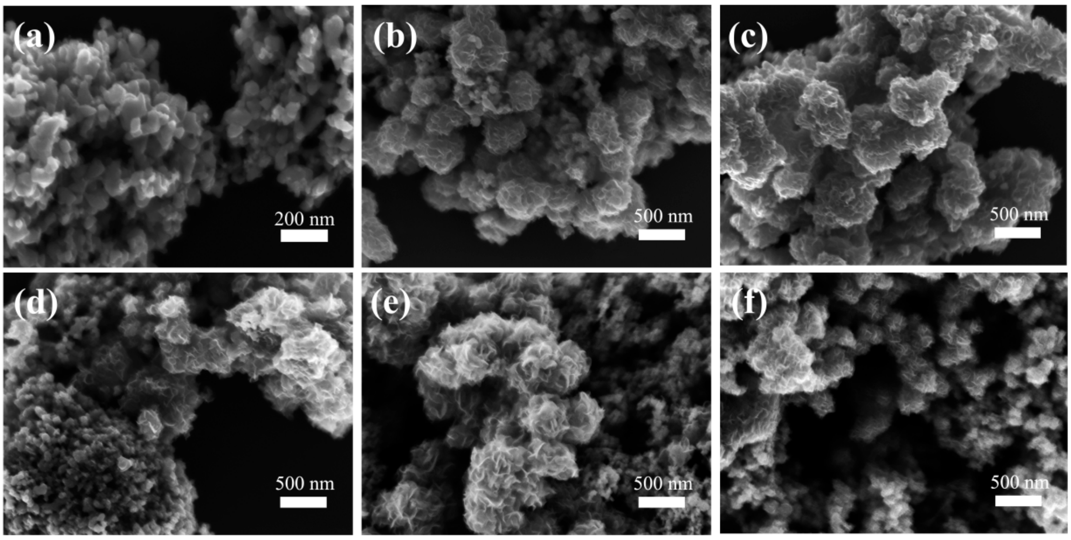

Figure S4. SEM images of (**a**) $NiSe_2$, (**b**) $MoS_2/NiSe_2$-1, (**c**) $MoS_2/NiSe_2$, (**d**) $MoS_2/NiSe_2$-2, (**e**) $MoS_2/NiSe_2$/rGO-1, and (**f**) $MoS_2/NiSe_2$/rGO-2.

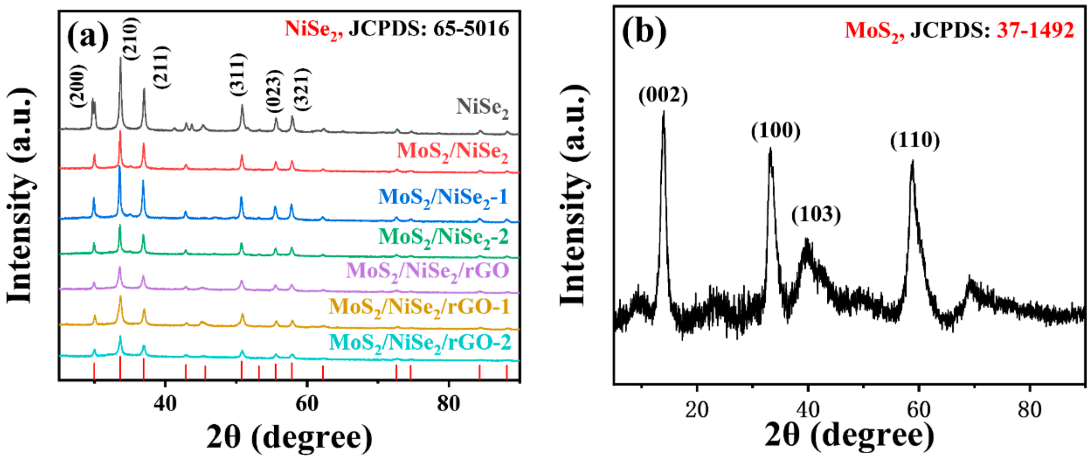

Figure S5. XRD patterns of (**a**) $NiSe_2$, $MoS_2/NiSe_2$, $MoS_2/NiSe_2$-1, $MoS_2/NiSe_2$-2, $MoS_2/NiSe_2$/rGO, $MoS_2/NiSe_2$/rGO-1, $MoS_2/NiSe_2$/rGO-2 and (**b**) the pure MoS_2.

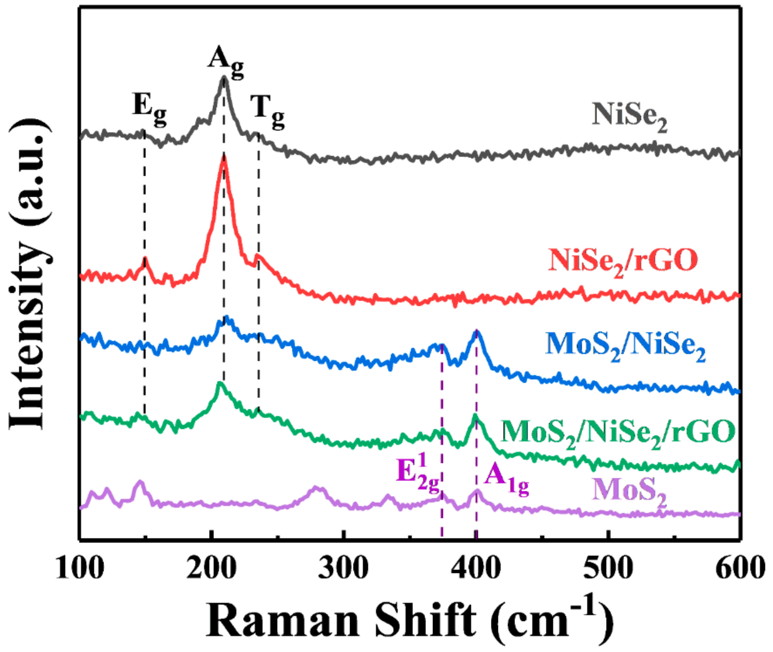

Figure S6. Raman spectra of $NiSe_2$, $NiSe_2$/rGO, $MoS_2/NiSe_2$, $MoS_2/NiSe_2$/rGO and MoS_2.

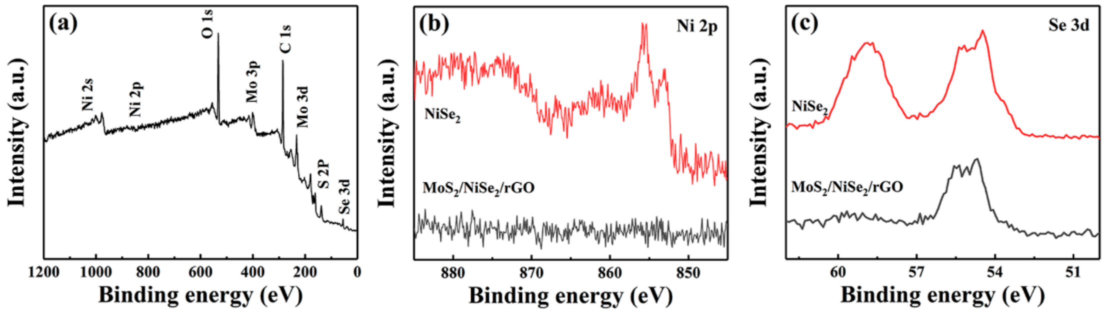

Figure S7. (**a**) XPS survey spectra. (**b**) Ni 2p and (**c**) Se 3d high resolution spectrum of $MoS_2/NiSe_2$/rGO, respectively.

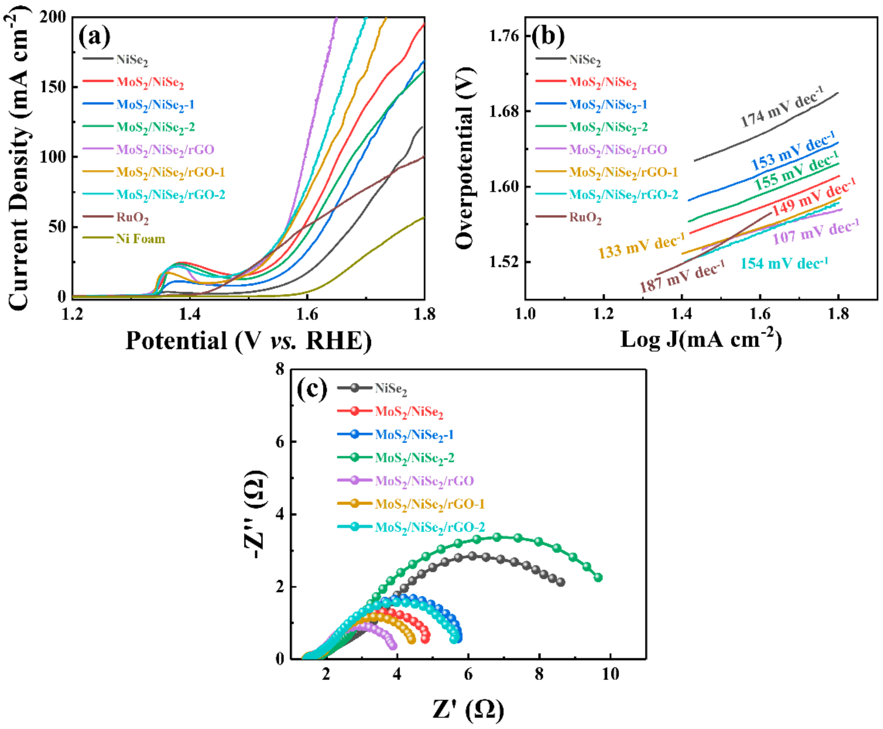

Figure S8. (**a**) Polarization curves (iR-corrected) at a scan rate of 2 mV s−1 of $NiSe_2$, $MoS_2/NiSe_2$, $MoS_2/NiSe_2$-1, $MoS_2/NiSe_2$-2, $MoS_2/NiSe_2$/rGO, $MoS_2/NiSe_2$/rGO-1, $MoS_2/NiSe_2$/rGO-2, RuO_2 and Ni foam, respectively. (The overall thickness, porosity, thread thickness, and pore density of Ni foam is 0.3 mm, 96%, 40–100 μm, and 110 PPI, respectively.) (**b**) Tafel plots of a series of samples. (**c**) Nyquist plots at an overpotential of 300 mV.

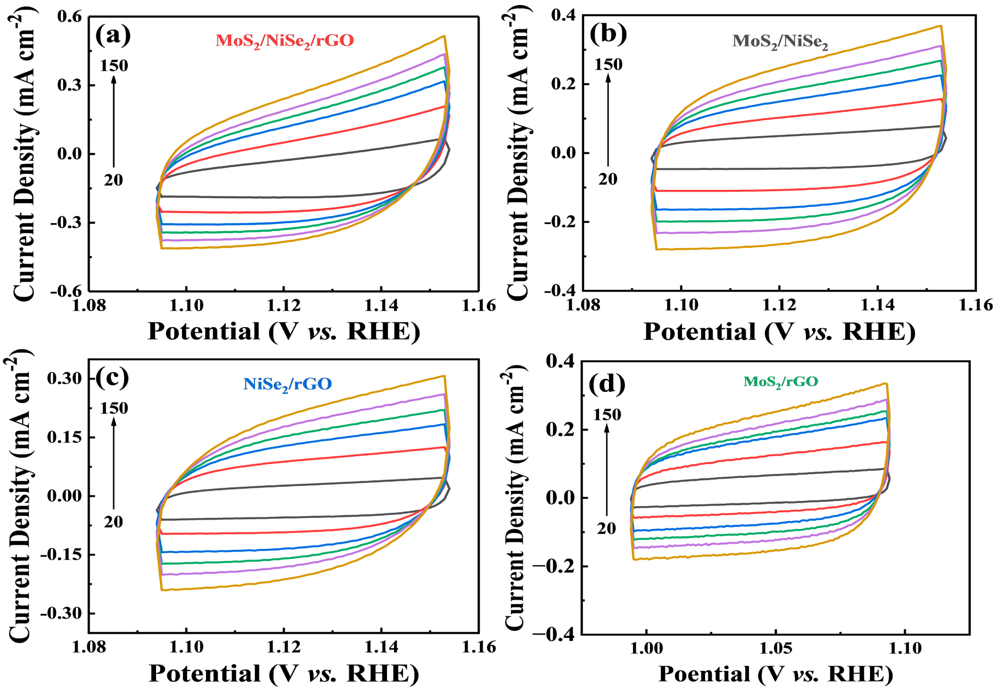

Figure S9. CV curves for (a) $MoS_2/NiSe_2$/rGO, (b) $MoS_2/NiSe_2$, (c) $NiSe_2$/rGO, (d) MoS_2/rGO with various scan rates (20, 50, 80, 100, 120, 150 mV S^{-1}).

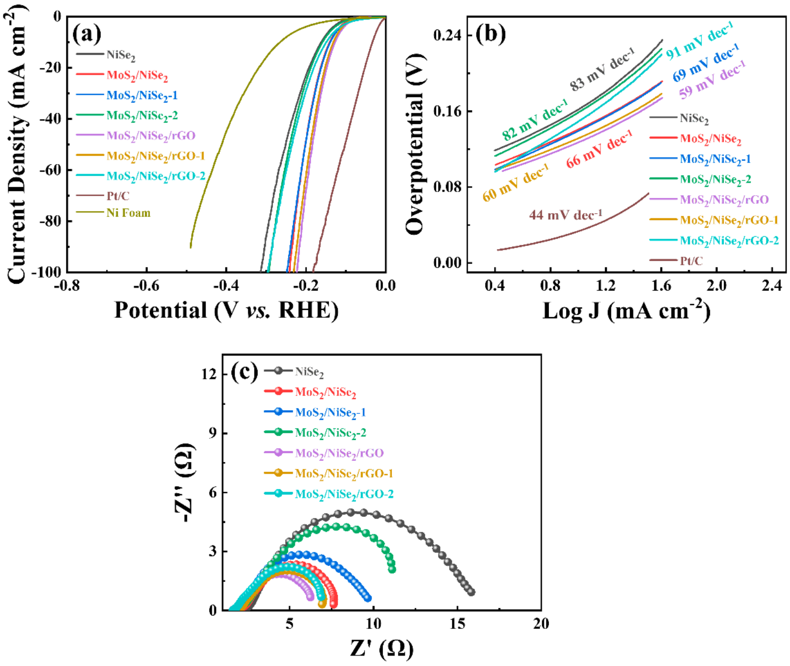

Figure S10. (**a**) Polarization curves (iR-corrected) at a scan rate of 5 mV s^{-1} of $NiSe_2$, $MoS_2/NiSe_2$, $MoS_2/NiSe_2$-1, $MoS_2/NiSe_2$-2, $MoS_2/NiSe_2$/rGO, $MoS_2/NiSe_2$/rGO-1, $MoS_2/NiSe_2$/rGO-2, Pt/C and Ni foam, respectively. (**b**) Tafel plots of a series of samples. (**c**) Nyquist plots at an overpotential of 120 mV.

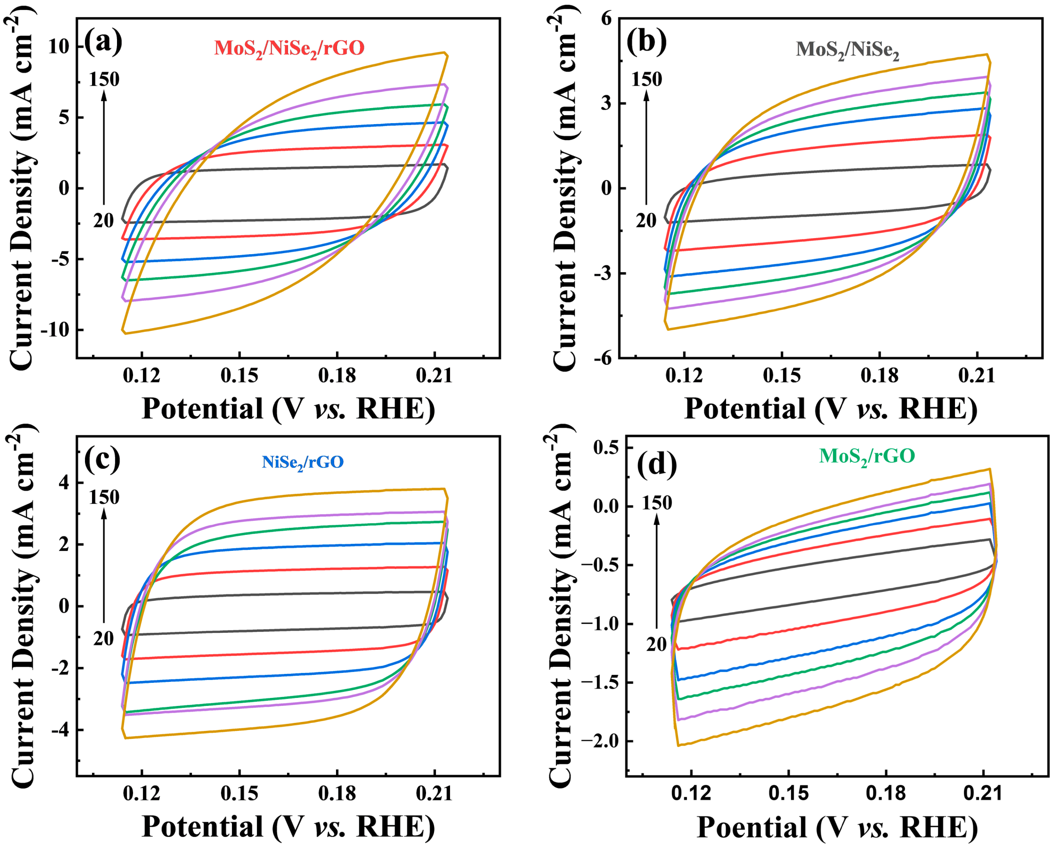

Figure S11. CV curves for (**a**) $MoS_2/NiSe_2$/rGO, (**b**) $MoS_2/NiSe_2$, (**c**) $NiSe_2$/rGO, (**d**) MoS_2/rGO with various scan rates (20, 50, 80, 100, 120, 150 mV S^{-1}).

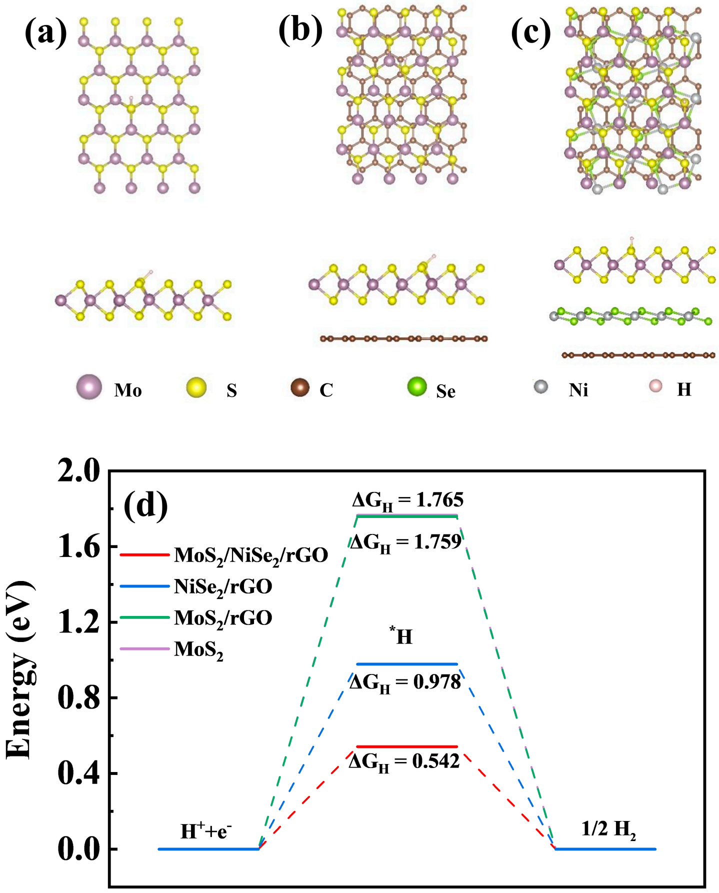

Figure S12. Optimized structural representations for hydrogen adsorption at (**a**) MoS_2, (**b**) MoS_2/rGO, (**c**) $MoS_2/NiSe_2$/rGO. Calculated free energy diagrams of the HER on MoS_2, MoS_2/rGO, $NiSe_2$/rGO, and $MoS_2/NiSe_2$/rGO, respectively.

DFT Calculation method: The present first-principles density-functional theory (DFT) calculations were performed using the Vienna ab initio simulation package (VASP)[S1]. The generalized gradient approximation (GGA) with revised-Perdew−Burke−Ernzerhof (RPBE) was applied for the exchange−correlation functionals[S2]. The wave functions were employed in a plane wave basis set with an energy cutoff of 400 eV. The convergence criterion for the self-consistency process was set to 10^{-4} eV between two ionic steps. During the structural relaxations, all atoms are fully relaxed until the residual forces on each direction are smaller than 0.02 eV/Å. The zero damping DFT-D3 method of Grimme[S3] was adopted in describing van der Waals interactions. To minimize the lattice mismatch, the periodic supercell slab models were constructed among a $4\times6\sqrt{3}$ MoS_2, a 2×3 $NiSe_2$ and a $6 \times 8\sqrt{3}$ graphene monolayer with a $2 \times 2 \times 1$ k-point mesh, All the slab models were separated by a vacuum of 15 Å thickness to ensure decoupling between neighboring slabs.

The Gibbs free energy change for each elemental step of the HER was calculated based on the computational hydrogen electrode (CHE) model[S4] proposed by Nørskov et al., expressed as:

$$\Delta G = \Delta E + \Delta E_{ZPE} - T\Delta S \tag{1}$$

Where ΔE, ΔE_{ZPE}, and ΔS are the energy obtained directly from DFT calculations, change in zero-point energy, and change in entropy terms, respectively, with temperature T=298.15 K.

Table S1. Comparison of electrocatalytic performance of different catalysts in 1 M KOH electrolyte.

Catalyst	Loading* (mg cm^{-2})	OER	HER	Overall	Ref.
		Overpotential @ j (mV@10 mA cm^{-2})		Voltage (V@10 mA cm^{-2})	
$MoS_2/NiSe_2$/rGO	4	230	127	1.52	This work
Ni–Co–P hollow nanobricks	2	270	107	1.62	S5
$Fe_{0.09}Co_{0.13}$-$NiSe_2$/CFC	--	251	92	1.52	S6
$NiSe_2$/Ni foam	0.2	235	166	1.64	S7
Ni_3S_2@NGCLs/NF	--	271	140	1.55	S8
NiMo-PVP	0.5–0.6	297	147	1.66	S9
N-$NiMoO_4/NiS_2$	0.2	283	99	1.60	S10

* The comparison catalysts are all non-precious metals, and this table can be used to measure the catalytic performance of the different catalysts without considering the small cost gap caused by the different loadings.

Table S2. Comparison of the electrocatalytic activity of $MoS_2/NiSe_2$/rGO with MoS_2/rGO, $NiSe_2$/rGO in 1 M KOH electrolyte.

Catalyst	OER / mV			HER / mV		
	η_{20}	η_{50}	η_{100}	η_{10}	η_{50}	η_{100}
$MoS_2/NiSe_2$/rGO	277	335	367	127 mV	184	223
$MoS_2/NiSe_2$	299	363	423	141	202	241
$NiSe_2$/rGO	328	365	407	164	247	301
MoS_2/rGO	390	460	490	259	346	374

Reference

1. [S1] Kresse, G.; Furthmüller, J., Efficient iterative schemes for ab initio total-energy calculations using a plane-wave basis set. *Phys. Rev. B* **1996**, *54*, 11169–11186.
2. [S2] Hammer, B.; Hansen, L.; Nørskov, J., Improved adsorption energetics within density-functional theory using revised Perdew-Burke-Ernzerhof functionals. *Phys. Rev. B* **1999**, *59*, 7413–7421.
3. [S3] Grimme, S., Semiempirical GGA-type density functional constructed with a long-range dispersion correction. *J. Comput. Chem.* **2006**, *27*, 1787–1799.
4. [S4] Nørskov, J.; Bligaard, T.; Logadottir, A.; Kitchin, J.; Chen, J.; Pandelov, S.; Stimming, U., Trends in the exchange current for hydrogen evolution. *Phys. Inorg. Chem.* **2005**, *152*, J23–J26.
5. [S5] Hu, E.; Feng, Y.; Nai, J.; Zhao, D.; Hu, Y.; Lou, X., Construction of hierarchical Ni–Co–P hollow nanobricks with oriented nanosheets for efficient overall water splitting. *Energy Environ. Sci.*, **2018**, *11*, 872–880.
6. [S6] Sun, Y.; Xu, K.; Wei, Z.; Li, H.; Zhang, T.; Li, X.; Cai, W.; Ma, J.; Fan, H.; Li, Y., Strong electronic interaction in dual-cation-incorporated $NiSe_2$ Nanosheets with lattice distortion for highly efficient overall water splitting. *Adv. Mater.* **2018**, *30*, 1802121.
7. [S7] Zhang, J.; Wang, Y.; Zhang, C.; Gao, H.; Lv, L.; Han, L.; Zhang, Z., Self-supported porous $NiSe_2$ nanowrinkles as efficient bifunctional electrocatalysts for overall water splitting. *ACS Sustainable Chem. Eng.* **2017**, *6*, 2231–2239.
8. [S8] Li, B.; Li, Z.; Pang, Q.; Zhang, J., Core/shell cable-like Ni_3S_2 nanowires/N-doped graphene-like carbon layers as composite electrocatalyst for overall electrocatalytic water splitting. *Chem. Eng. J.* **2020**, *401*,126045.
9. [S9] Zhang, Y.; Xia, X.; Cao, X.; Zhang, B.; Tiep, N.; He, H.; Chen, S.; Huang, Y.;Fan, H., Ultrafine metal nanoparticles/N-doped porous carbon hybrids coated on carbon fibers as flexible and binder-free water splitting catalysts. *Adv. Energy Mater.* **2017**, *7*, 1700220.
10. [S10] An, L.; Feng, J.; Zhang, Y.; Wang, R.; Liu, H.; Wang, G.; Cheng, F.; Xi, P., Epitaxial heterogeneous interfaces on N-Ni-MoO_4/NiS_2 nanowires/nanosheets to noost hydrogen and oxygen production for overall water splitting. *Adv. Funct. Mater.* **2019**, *29* 1805298.
